# OKN and Pupillary Response Modulation by Gaze and Attention Shifts

**DOI:** 10.3390/jemr18020011

**Published:** 2025-04-07

**Authors:** Kei Kanari, Moe Kikuchi

**Affiliations:** 1 Advanced Institute of So-Go-Chi (Convergence Knowledge) Informatics, Tohoku University, 2-1-1 Katahira, Aoba-ku, Sendai 980-8577, Japan; 2 Department of Information and Communications Engineering, Institute of Science Tokyo, 4259 Nagatsuda-cho, Midori-ku, Yokohama 226-8502, Japan; 3 Department of Fundamental Engineering, Utsunomiya University, 7-1-2 Yoto, Utsunomiya 321-8585, Japan; 4 Child and Family Support Center, Meguro City Office, 2-19-15 Kamimeguro, Tokyo 153-8573, Japan; moekikuchi@gmail.com

**Keywords:** optokinetic nystagmus, pupil response, attention shifts

## Abstract

Pupil responses and optokinetic nystagmus (OKN) are known to vary with the brightness and direction of motion of attended stimuli, as well as gaze position. However, whether these processes are controlled by a common mechanism remains unclear. In this study, we investigated how OKN latency relates to pupil response latency under two conditions: gaze shifts (eye movement) and attention shifts (covert attention without eye movement). As a result, while OKN showed consistent temporal changes across both gaze and attention conditions, pupillary responses exhibited distinct patterns. Moreover, the results revealed no significant correlation between pupil latency and OKN latency in either condition. These findings suggest that, although OKN and pupillary responses are influenced by similar attentional processes, their underlying mechanisms may differ.

## 1. Introduction

The human visual system optimizes perception by dynamically adjusting both pupil size and eye movements. The pupil constricts in bright environments and dilates in dark environments to manage the light that enters the eye, thereby enhancing visual acuity [1,2,3]. Simultaneously, eye movements help maintain clear vision by stabilizing images on the retina [4,5]. One such mechanism is optokinetic nystagmus (OKN), which occurs in response to continuous motion in the visual field. OKN consists of a slow phase that tracks moving objects and a quick phase that resets the gaze in the direction opposite to the object’s motion [6]. This process helps to maintain a stable retinal image despite motion in the environment.

Beyond their reflexive responses to brightness and motion, both pupil size and OKN are influenced by attention. Attention can be directed to a location that differs from the current gaze position [7], and pupillary responses reflect not only the luminance at the point of fixation but also at the attended location. For example, the pupil dilates when attention is directed toward a dark object and constricts when attending to a bright object, even without a shift in gaze [8,9,10,11]. Similarly, OKN can be induced not only by motion at the gaze location but also by motion at an attended peripheral location. When stimuli with opposite motion directions are presented centrally and peripherally, OKN tends to align with the motion in the attended region [12,13,14,15,16].

Furthermore, emerging evidence suggests a common attentional mechanism underlying OKN and pupillary responses. During OKN, attention is known to shift in the direction of the quick phase, and the pupil responds to the brightness of the newly attended location [17]. Similarly, when observing transparent motion [18] or binocular rivalry stimuli [19], OKN varies with the motion direction of the dominant visual element, while pupil responses change in accordance with its brightness. These findings suggest that OKN and pupillary adjustments are both influenced by attentional processing; however, recent evidence highlights that they may involve distinct physiological systems, the visuomotor and autonomic nervous systems, respectively. This distinction is supported by neuroanatomical evidence showing that OKN and pupillary light reflexes originate in different brainstem structures [20,21].

In terms of neural substrates, the nucleus of the optic tract (NOT) in the pretectal area is known to play a central role in generating horizontal OKN, by detecting retinal slip and initiating compensatory eye movements [22,23]. Furthermore, recent neuroimaging and neurophysiological studies suggest that the slow-phase of OKN may recruit cortical visuomotor networks similar to those used in smooth pursuit, particularly under conditions involving voluntary attention to motion [20,24]. Meanwhile, the nucleus pretectalis olivaris (NPO), also located in the pretectal region, mediates the pupillary light reflex by integrating luminance signals and driving pupil constriction [25]. Together, these pretectal nuclei serve as essential nodes in the neural circuitry underlying reflexive visual responses to motion and luminance.

In addition to OKN, pupillary responses are influenced by saccade preparation. Saccades are quick eye movements that move the gaze from one location to another, typically following a covert shift in attention [26,27,28]. Before the eyes move, the pupil begins adjusting its size in response to the expected brightness at the upcoming fixation location [29], suggesting that the pupillary light response (PLR) is dynamically connected to both attention and the preparation for eye movements. Although the superior colliculus (SC) has been implicated in attentional control and gaze shifts [30,31,32], its direct involvement in reflexive OKN and pupillary light responses is likely limited compared to pretectal structures such as the NOT and NPO.

Building on this evidence, the current study aimed to investigate whether OKN and pupillary responses are governed by shared or distinct control processes under different attentional states. Specifically, we compared the response latencies of OKN and pupil size under both gaze shift and covert attention conditions. We hypothesized that if both systems were governed by a common attentional control mechanism, their latencies would be correlated. However, our findings revealed no such correlation in both conditions, suggesting that OKN and pupillary responses, though both modulated by attention, are controlled by separate neural systems.

## 2. Materials and Methods

### 2.1. Participants

A total of 28 students took part in this experiment (19 female; mean age: 19.2 years; age range: 18–27 years). They were naïve to the purpose of the study. The participant count was established based on a previous study [18] that simultaneously measured OKN and pupil responses. All participants had 20/20 vision or better, including those with corrected vision.

### 2.2. Stimuli

**Figure 1:**
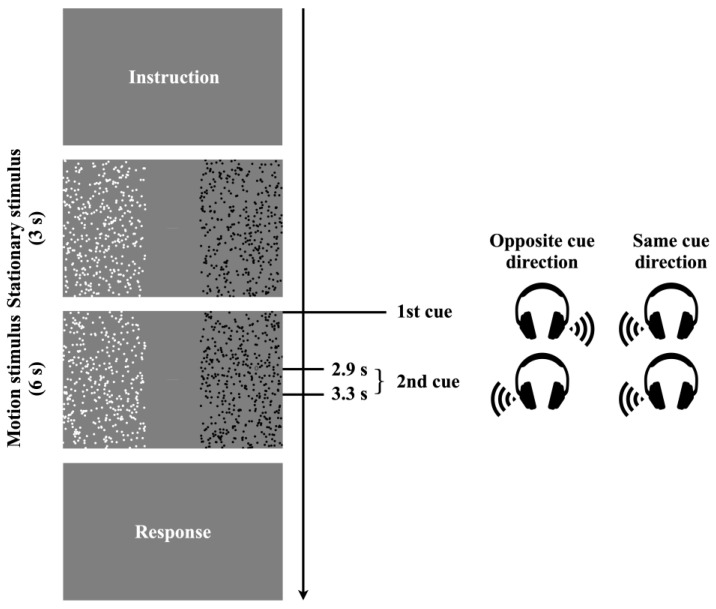
The flow of the experimental procedure in a trial. In each trial, participants directed either their gaze or covert attention toward a random dot pattern, as indicated by an auditory cue. Shortly after each cue, a target numeral (“0” or “1”) appeared within the cued motion stimulus, moving in the same direction and at the same speed as surrounding dots. Each trial included two auditory cues, leading to the appearance of two target numerals at different time points. After the motion ended, participants reported both numerals using a numeric keypad.

The motion stimuli are composed of a collection of randomly placed dots (Figure 1, third panel from the top). Each dot had a diameter of 0.54°. The luminance of the white and black dots was 32.06 and 0.04 cd/m^2^, respectively. The dot density within the stimulus was 2.58 dot/deg^2^, and the dots moved at a velocity of 11.36 deg/s. The black and white dots were always moving in opposite directions to each other. The size of the motion stimulus was 18.45 × 31.7°. The left and right motion stimuli were separated by 12°. To maintain participants’ gaze centered on the screen while allowing OKN parallel to the motion direction, we presented a fixation line (length: 2.7°; width: 0.19°; luminance: 8.43 cd/m^2^) at the center. The background was gray (6.86 cd/m^2^). A target (“0” or “1”) was introduced to direct attention to the cued area. The target, with a height of 1.35°, moved at the same velocity and in the same direction as the dots. It appeared within the motion stimulus area, specifically within 12.2° above and below the center, and emerged from the edge of the area. The direction of attention or gaze was cued by stereo sound consisting of two 120 bpm beeps. Figure 1 illustrates the trial flow.

### 2.3. Procedure

At the start of the experiment, a nine-point eye-tracker calibration was performed. Each trial began with a central fixation line and stationary black and white dots displayed for 3 s, during which participants were instructed to fixate on the line. After this period, the fixation line disappeared and the dots began to move for 6 s.

At the onset of the motion, a first auditory cue (1st cue) was presented to indicate the direction of attention or gaze. In the “attention condition”, participants were instructed to maintain central fixation while covertly attending to the motion stimulus in the cued direction. In the “gaze condition”, participants were instructed to shift their gaze toward the cued motion stimulus.

A target numeral (“0” or “1”) appeared once in the cued field for 0.83 s, at a random time between 1.66 and 2.16 s after motion onset. Then, a second auditory cue (2nd cue) was presented between 2.9 and 3.3 s, followed by a second target in the same field (again for 0.83 s), appearing between 4.66 and 5.16 s.

After the motion ended, participants were reported to enter the two numerals they observed using a numeric keypad. Only trials in which both responses were correct were included in the analysis. After each response, feedback was given indicating whether the answers were correct or incorrect. Participants were allowed to rest freely between trials while keeping their head positioned on the chin rest. There were no constraints on the duration of these breaks. In practice, most participants completed each condition (3 blocks = 48 trials) within approximately 12 min. The attention and gaze conditions were conducted in separate sessions, with most participants taking a short break of about 2 min between sessions.

Each block consisted of 16 trials, incorporating a randomized combination of two motion directions (rightward or leftward), two dot brightness levels (white or black), two attention directions (left or right), and two cue directions (same or opposite). Each participant completed three blocks in each condition (attention condition or gaze condition), totaling 48 trials (16 × 3) per condition. The gaze and attention conditions were conducted separately, leading to a total of 96 trials (48 × 2).

### 2.4. Apparatus

The stimuli were displayed on a 24.1-inch LCD screen (EIZO CS2420, EIZO Corporation, Ishikawa, Japan) with a resolution of 1920 × 1200 pixels and a refresh rate of 60 Hz. The experiment was performed in a darkroom. The participants had their heads stabilized using a forehead and chin rest while they listened to the audio cues through wireless earphones (Apple AirPods Pro, Apple Inc., Cupertino, CA, USA). The viewing distance from the eyes to the display was 57 cm. The stimuli were created using MATLAB R2022b (MathWorks, Inc., Natick, MA, USA) along with the Psychophysics Toolbox [33,34] on a MacBook Pro (Apple Inc., CA, USA). Participants’ pupil size and right-eye position were measured at a sampling rate of 500 Hz using an infrared camera (iRecHS2 Ver.660) [35]. The system’s optimal accuracy was 0.028° for the horizontal position tracking and 0.096° for the vertical position tracking.

### 2.5. Analysis

Only trials in which participants correctly identified both target numbers were considered for analysis. In the gaze condition, the average probability of correctly identifying both targets was 93.82%, whereas in the attention condition, it was 56.99%. Blink data were detected using the iRecHS2 algorithm and treated as missing data. Pupil data were smoothed using a Gaussian-weighted moving average filter (window size: 100). Pupil size during the stimuli presentation (9 s) in each trial was normalized to z-scores [19,36,37].

The time variation in the slow-phase velocity of OKN was determined with partial reference to the methods used in previous studies [18,19,37]. First, as part of data preprocessing, values greater than 100,000 and values of 0 were replaced with NaN to handle outliers and invalid data. Then, an 11-point moving average was applied to the eye position data to smooth the values. Next, the velocity of eye movement was calculated by taking the time derivative of the position data, and these velocity data were also smoothed using a moving average with a window size of 11. Using the smoothed velocity data, saccades were detected. Since the stimulus velocity was 11.36 deg/s, peaks and troughs where the velocity exceeded ±20 deg/s were identified. Then, the points where the velocity reached a local minimum before and after each peak were determined, and the range between these two points was defined as the saccadic phase. Saccade removal was performed by replacing the data within the identified range with NaN. These NaN values were then linearly interpolated to reconstruct the slow-phase velocity trajectory, under the assumption that the eye would have continued its smooth motion in the absence of saccades. This procedure follows established methods in previous studies on OKN and smooth pursuit [19,37,38,39], in which fast phases are removed based on velocity thresholds and the missing intervals are filled using linear interpolation. Finally, the data were smoothed using a Gaussian-weighted moving average filter with a window size of 1000. This process effectively removed the influence of saccades while reconstructing a natural representation of the OKN slow-phase velocity data. For each participant, OKN and pupil data were separately averaged across trials under the same experimental conditions. As a result, each participant had a single averaged time-series dataset for OKN and pupil responses for each condition.

The latency of OKN and pupil responses during gaze or attentional shifts was determined based on the method described in a previous study [40]. First, velocity and acceleration were calculated from the smoothed slow-phase velocity and pupillary size, and the acceleration was further smoothed with a Gaussian-weighted filter (window size: 500). Then, local minima (valleys) in the acceleration were detected, and the lowest valley within ±1.5 s of the second auditory cue presentation (set as time 0) was identified. The time of this minimum acceleration was defined as the latency.

## 3. Results

### 3.1. Slow-Phase Velocity of OKN

**Figure 2:**
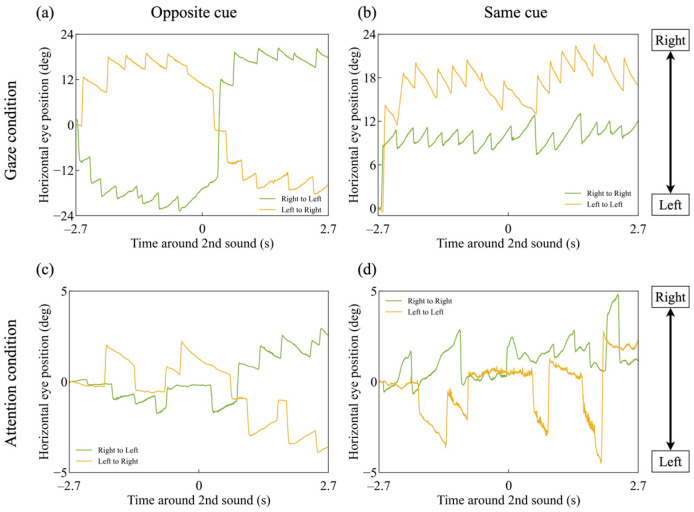
Time-series changes in horizontal eye position in a representative single trial under (**a**) the gaze-opposite cue condition, (**b**) the gaze-same cue condition, (**c**) the attention-opposite cue condition, and (**d**) the attention-same cue condition.

Figure 2 illustrates a representative example of raw horizontal eye movement data from a typical participant. The upper and lower panels display the horizontal eye position in the gaze and attention conditions, respectively. The right and left panels correspond to the opposite cue and same cue conditions, respectively. The horizontal axis depicts time, with 0 s indicating the display of the second cue. The vertical axis represents horizontal eye position (in degrees), with positive values indicating rightward eye movements. The legend indicates the motion direction of the dots that were either gazed at or attended to.

Figure 2a shows that directing the gaze toward a leftward-moving random dot pattern induces OKN, characterized by leftward slow phases that follow the motion of the dots and rightward quick phases that reset gaze direction. Similarly, the green trace in Figure 2a and the traces in Figure 2b also demonstrate that OKN occurs in the direction corresponding to the motion of the random dot pattern toward which the gaze is directed.

Although the amplitude and frequency of OKN are reduced in the attention conditions (Figure 2c,d), the overall pattern remains similar to that observed in the gaze conditions.

**Figure 3:**
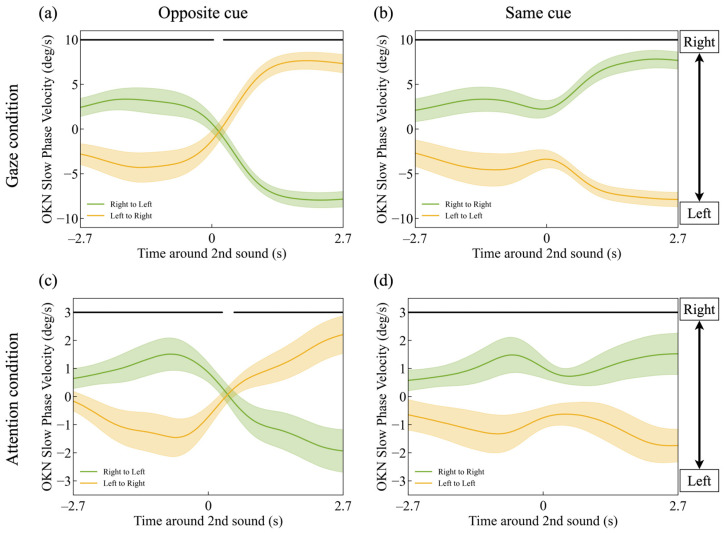
Time-series changes in OKN slow-phase velocity for (**a**) the gaze-opposite cue condition, (**b**) the gaze-same cue condition, (**c**) the attention-opposite cue condition, and (**d**) the attention-same cue condition. The horizontal black bars mark the data points where the two data lines show a significant difference, corresponding to an expected FDR of 0.05.

Figure 3 shows the mean velocity of the OKN slow-phase across all participants. Except that the vertical axis represents the slow-phase velocity of OKN, the graphs can be interpreted in the same way as Figure 2. The vertical axis represents the velocity of the OKN slow-phase (deg/s), where positive values indicate rightward eye movement. The lightly shaded area represents the 95% confidence interval of the participants’ mean values. The data were analyzed using the Benjamini–Hochberg procedure to control the false discovery rate (FDR) [41], following Einhäuser et al. [36]. This method is independent of the sampling rate. The horizontal black lines mark the time points where the two traces show a significant difference, with a false discovery rate (FDR) of 0.05 (Figure 3a: *t*-test, *p* < *p*_FDR=0.05_, with a threshold of *p*_thresh,FDR=0.05_ = 0.047186. Figure 3b: *t*-test, *p* < *p*_FDR=0.05_, with a threshold of *p*_thresh,FDR=0.05_ = 0.000017. Figure 3c: *t*-test, *p* < *p*_FDR=0.05_, with a threshold of *p*_thresh,FDR=0.05_ = 0.047024. Figure 3d: *t*-test, *p* < *p*_FDR=0.05_, with a threshold of *p*_thresh,FDR=0.05_ = 0.000739).

The green line in Figure 3a shows that the mean velocity of the OKN slow-phase corresponded to the motion direction of the directly gazed-at dots. Positive (rightward) OKN slow-phase velocity occurred when the first cue was presented in the direction of the rightward-moving dots, whereas negative (leftward) velocity occurred when the second cue was presented in the direction of the leftward-moving dots. Similarly, as shown by the yellow line in Figure 3a and the green and yellow lines in Figure 3b, the OKN slow-phase velocity aligned with the motion direction of the directly gazed-at dots. The OKN slow-phase velocity decreased before the second cue was presented.

Figure 3c,d indicates that the mean OKN slow-phase velocity in the attention condition followed a similar pattern to that in the direct gaze condition, except that the velocity was lower than in the direct gaze condition.

### 3.2. Pupil Response

**Figure 4:**
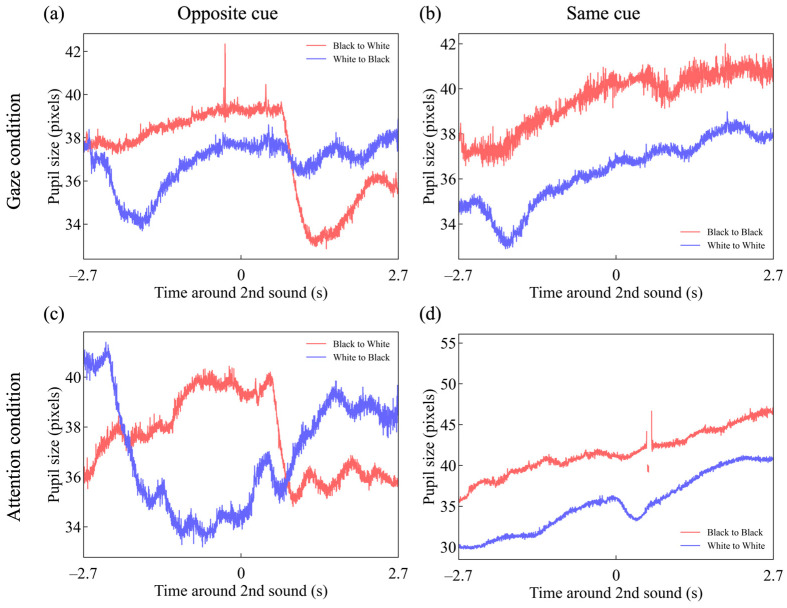
Time-series changes in pupil size in a representative single trial under (**a**) the gaze-opposite cue condition, (**b**) the gaze-same cue condition, (**c**) the attention-opposite cue condition, and (**d**) the attention-same cue condition.

Figure 4 presents raw pupil size data from a single trial in each condition, taken from a representative participant. Each panel corresponds to the same conditions as those shown in Figure 2 and Figure 3. The vertical axis indicates pupil size, measured in pixels based on recordings from the eye-tracking camera. Each legend denotes the brightness of the dots that were either gazed at or attended to. Although pupil size is expressed in pixels and thus cannot be directly compared across participants, clear temporal modulations in response to the presented stimuli can be observed. For group-level analyses, the pupil size data were standardized using z-score transformation.

As shown by the red trace in Figure 4a, pupil size varied in accordance with the brightness of the dots toward which the gaze was directed. Specifically, when the gaze was initially directed toward a black dot, the pupil gradually dilated; after the sound was presented and the gaze shifted to a white dot, the pupil constricted. A similar pattern was observed in the attention condition (Figure 4c). However, in the same cue conditions, where the gaze or attention remained fixed, the pupil tended to continue dilating throughout the trial regardless of the brightness condition.

**Figure 5:**
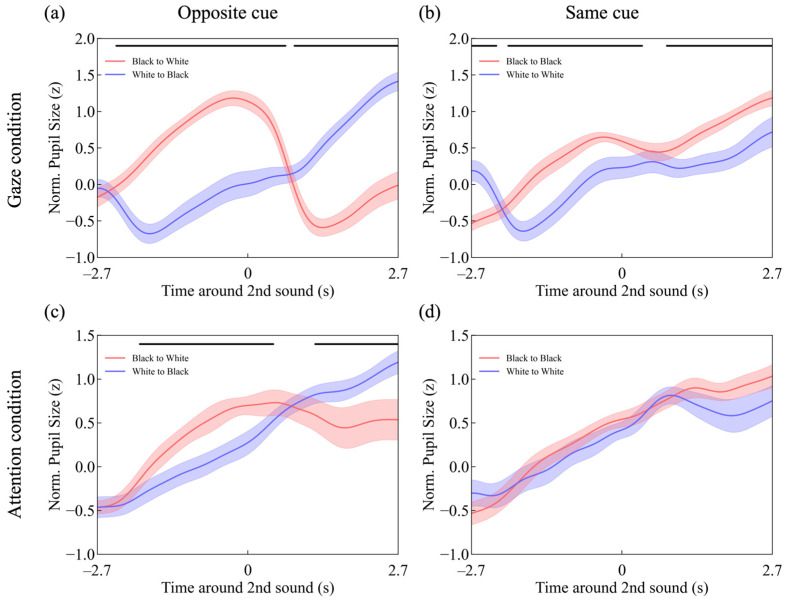
Time-series changes in pupil size for (**a**) the gaze-opposite cue condition, (**b**) the gaze-same cue condition, (**c**) the attention-opposite cue condition, and (**d**) the attention-same cue condition. The horizontal black bars mark the data points where the two data lines show a significant difference, corresponding to an expected FDR of 0.05.

Figure 5 shows the mean pupillary response of all participants. The vertical axis represents pupil size normalized to z-scores for each trial during the 9 s stimulus presentation period. The horizontal black bars mark the data points where the two data lines show a significant difference, with a false discovery rate (FDR) of 0.05 (Figure 3a: *t*-test, *p* < *p*_FDR=0.05_, with a threshold of *p*_thresh,FDR=0.05_ = 0.044448. Figure 3b: *t*-test, *p* < *p*_FDR=0.05_, with a threshold of *p*_thresh,FDR=0.05_ = 0.043941. Figure 3c: *t*-test, *p* < *p*_FDR=0.05_, with a threshold of *p*_thresh,FDR=0.05_ = 0.035967).

The red line in Figure 5b shows a similar pupillary response to luminance under the direct gaze condition. However, as shown by the blue line in Figure 5b, when participants continuously gazed at white dots in the same area after the first cue, the pupil initially contracted but then gradually returned to baseline, continuing this trend after the second cue. Additionally, as seen in the red lines of Figure 5a,b, pupil size began to constrict before the presentation of the second cue.

Figure 5c,d shows that pupillary responses in the attention condition were similar to those in the direct gaze condition. However, in the attention condition, there was no clear pupillary constriction in response to the white dots attended to after the first cue, and no pupillary constriction was observed before the second cue.

### 3.3. Latency in Gaze and Attentional Shift

**Figure 6:**
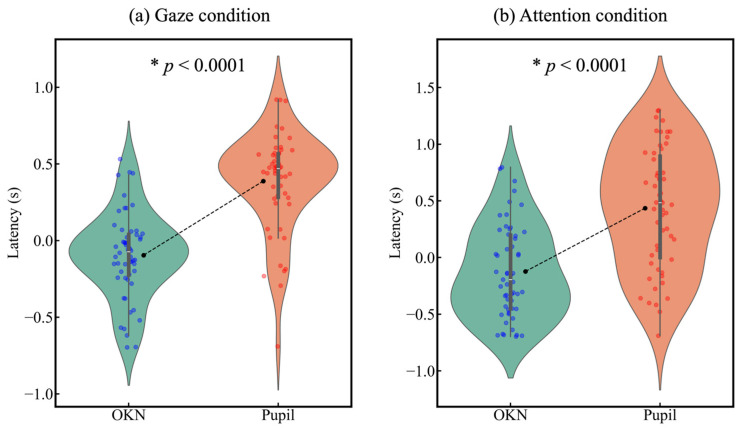
Violin plot of latency in (**a**) the gaze condition and (**b**) the attention condition. The black dot represents the mean, the thin gray line indicates the lower adjacent and upper adjacent values, and the thick gray bar represents the range between the 25th and 75th percentiles, with the white horizontal line inside indicating the median.

Considering the findings on temporal changes in pupillary size, an evident shift induced by the second auditory cue appeared under the black–white condition with the opposite cue. Therefore, we analyzed the relationship between OKN and pupil latency in this condition. The mean latencies of OKN and pupil responses showed a significant difference in both the gaze condition (*t*(52) = −6.626, *p* < 0.0001) and the attention condition (*t*(52) = −7.797, *p* < 0.0001) (Figure 6).

**Figure 7:**
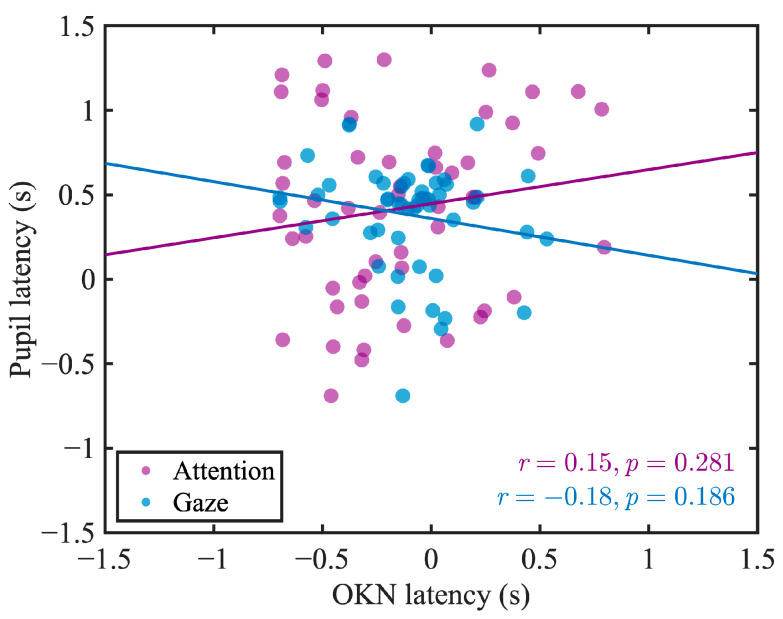
Scatter plots of OKN and pupil latency in the gaze condition (blue dots) and the attention condition (purple dots). The lines represent the linear regression fits for each scatter plot.

To examine the shared mechanisms of OKN and pupil responses in gaze and attention shifts, we analyzed the correlation between OKN latency and pupil latency. As a result, no significant correlation was found between OKN latency and pupil latency in either the gaze condition (*r* = −0.18, *p* = 0.186) or the attention condition (*r* = 0.15, *p* = 0.281) (Figure 7).

**Figure 8:**
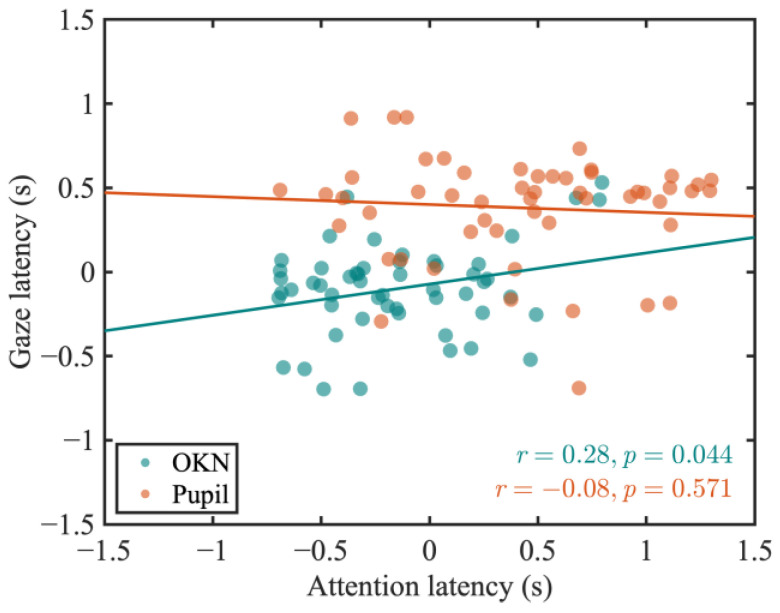
Scatter plots of latency in the gaze condition and attention condition for OKN (green dots) and pupil (orange dots). The lines represent the linear regression fits for each scatter plot.

To examine the shared control mechanism of gaze and attention shifts, we analyzed the correlation of latencies between the gaze and attention conditions for both OKN and pupil responses. As a result, no significant correlation was found for pupil latencies between the gaze and attention conditions (*r* = −0.08, *p* = 0.571). However, a significant correlation was observed for OKN latencies (*r* = 0.28, *p* = 0.044) (Figure 8).

## 4. Discussion

### 4.1. OKN Modulation by Gaze and Attention

In this study, we examined the time-series changes in OKN and pupil responses when gaze or attention was spatially shifted. Our results showed that in the attention condition, OKN occurred in the direction corresponding to motion, similar to the gaze condition. The significant correlation between OKN slow-phase velocity latency in the gaze and attention conditions further suggests that OKN shares a common control mechanism for both gaze and attention shifts. This result aligns with other studies’ findings, indicating that OKN is induced by covert attentional shifts alone [12,13,14,15,16], and is consistent with a recent study reporting that torsional OKN (tOKN) gain and latency are modulated by visual attention even under central gaze fixation [42]. Although tOKN is elicited by rotational motion and differs from horizontal OKN in directional characteristics, it shares fundamental visuomotor mechanisms, including attentional modulation of the velocity storage system.

Additionally, the attention condition showed a reduction in the slow-phase velocity of OKN relative to the gaze condition. This finding aligns with previous studies [12,13,15], suggesting that the central retina plays a significant role in human OKN [43]. Since peripheral vision provides weaker motion signals than central vision, the reduction in OKN velocity in the attention condition may reflect a difference in retinal input strength. This also supports the view that foveal stimulation enhances OKN gain, whereas covertly attending to peripheral stimuli yields weaker responses, even when attention is actively directed.

### 4.2. Pupillary Modulation by Gaze and Attention

Pupillary responses exhibited distinct temporal and amplitude characteristics compared to OKN. While some pupil responses in the attention condition appeared to reflect the brightness of the attended location, overall response patterns deviated markedly from the patterns observed in the gaze condition. Notably, no significant correlation was found between the latency of OKN slow-phase velocity and pupil latency in both the gaze and attention condition. This dissociation suggests that although both responses are modulated by attentional mechanisms, they are governed by fundamentally different physiological control systems, namely, the visuomotor and the autonomic nervous systems, respectively [20,21].

The study also revealed that pupil constriction in response to white dots was observed after the initial auditory cue presentation in the gaze condition but was absent in the attention condition (Figure 5a–d, blue line). One possible explanation for this difference is the increased cortical activity due to cognitive load and attentional control, which may suppress parasympathetic nervous system activity [44,45,46,47], leading to pupillary dilation under the attention condition.

Additionally, similar to OKN, pupil responses were generally weaker in the attention condition relative to the gaze condition. This finding aligns with previous studies [8,11] and may be associated with the fact that pupillary size varies according to the stimulus location [48,49]. For example, in the condition where the gaze shifted from a black dot to a white dot (Figure 5a, red line), a clear pupil response peak (trough) was observed around 0 and 1 s. However, in the attention condition, no such clear peak (trough) was observed. Pupil responses induced by peripheral stimuli tend to have smaller amplitudes, while those induced by foveal stimuli are larger [50]. Since the attention condition involved attending to stimuli in peripheral vision, the pupil response was weaker compared to the gaze condition.

### 4.3. Distinct Control Mechanisms for OKN and Pupillary Responses

A major conclusion of our research is that there was no significant correlation between the latency of OKN slow-phase velocity and pupil latency in both the gaze and attention conditions (Figure 7). This suggests that while both responses are influenced by attentional mechanisms, they are governed by distinct control mechanisms. Additionally, while the latency of OKN slow-phase velocity showed a significant correlation between the gaze and attention conditions (Figure 8, green dots), pupil latency did not exhibit such a correlation (Figure 8, orange dots).

These findings indicate that different mechanisms underlie pupil control in the gaze and attention conditions. In the gaze condition, pupil changes may be influenced by the pre-saccadic shift in attention or by luminance variations in the central retina accompanying gaze movements. In contrast, in the attention condition, various factors such as attentional control and cognitive load from the attention task may modulate pupillary responses [44,45,46,47]. This interpretation aligns with previous findings, showing that SC activity can influence pupil size independently of saccades [51,52]. However, such effects may reflect modulatory roles in attention-related arousal, rather than direct reflexive control. In contrast, the pretectal olivary nucleus (NPO) is thought to serve as the primary structure mediating pupillary light reflexes [25].

### 4.4. Relationship Between OKN Slow-Phase and Smooth Pursuit

The smooth pursuit system has been demonstrated to function in a manner closely resembling that of the saccadic system [53]. Nonetheless, the distinction between the slow-phase component of OKN and smooth pursuit eye movements remains unclear. Given that OKN slow-phase latency was significantly correlated between the gaze and attention conditions, it is possible that OKN and smooth pursuit share common control mechanisms related to both gaze and attention shifts.

Recent proposals suggest that the neurophysiology of pursuit eye movements may be divided into motion-correcting and position-correcting neural pathways, which has implications for visual processing during pursuit tasks [24]. The motion-correcting pathway may involve areas also responsible for the initiation of ocular function following responses and the slow-phase of OKN, such as the nucleus of the optic tract. Furthermore, it was proposed that these two pathways may interact depending on stimulus properties such as target size. However, it remains unclear whether this transition is governed by different attentional mechanisms or by a unified model based on the salience of target features.

These recent perspectives support the view that the slow-phase component of OKN, particularly under attentional modulation, may reflect shared visuomotor control mechanisms with the smooth pursuit system. This is further supported by fMRI findings showing overlapping neural activation patterns during both smooth pursuit and OKN, especially for look-nystagmus, which involves intentional visual tracking and evokes cortical oculomotor activity similar to pursuit movements [20]. Notably, attentional engagement appears to transform OKN from a reflexive response into a more volitional, pursuit-like behavior, recruiting higher-order cortical networks typically associated with smooth pursuit. Therefore, attentional modulation may serve as a key factor in linking the neural mechanisms of OKN and pursuit.

Nonetheless, additional research is required to clarify the extent of functional and anatomical overlap between these systems and to identify how attentional demands influence their respective dynamics.

### 4.5. Limitations

This study examined differences in OKN and pupillary responses using specific visual stimuli and attention manipulations (e.g., gaze shifts vs. attentional shifts). However, it did not explore other types of attention (e.g., spatial vs. feature-based attention) or different visual stimuli (e.g., dynamic vs. static stimuli, varying contrast conditions). Comparing OKN and pupillary responses under different types of attention and visual stimuli may provide deeper insights into the underlying mechanisms.

Although prior studies have suggested the potential involvement of subcortical structures (e.g., superior colliculus), the present study did not aim to localize the underlying neural substrates. Future studies using neuroimaging or electrophysiological methods are warranted to delineate the distinct neural circuits underlying OKN and pupillary responses under attentional modulation.

## Data Availability

The original data presented in the study are openly available in GitHub at https://doi.org/10.5281/zenodo.14934575 (accessed on 11 March 2025).

## References

[1] Campbell F.W., Gregory A.H. (1960). Effect of size of pupil on visual acuity. Nature.

[2] Woodhouse J.M. (1975). The effect of pupil size on grating detection at various contrast levels. Vis. Res..

[3] Mathôt S. (2018). Pupillometry: Psychology, physiology, and function. J. Cogn..

[4] Walls G.L. (1962). The evolutionary history of eye movements. Vis. Res..

[5] Land M.F. (1999). Motion and vision: Why animals move their eyes. J. Comp. Physiol. A.

[6] Purkinje J. (1825). Beobachtungen und Versuche zur Physiologie der Sinne Neue Beitrage zur Kenntnis des Sehens in Subjektiver Hinsicht.

[7] Posner M.I. (1980). Orienting of attention. Q. J. Exp. Psychol..

[8] Binda P., Pereverzeva M., Murray S.O. (2013). Attention to bright surfaces enhances the pupillary light reflex. J. Neurosci..

[9] Mathôt S., Van Der Linden L., Grainger J., Vitu F. (2013). The pupillary light response reveals the focus of covert visual attention. PLoS ONE.

[10] Binda P., Murray S.O. (2015). Spatial attention increases the pupillary response to light changes. J. Vis..

[11] Binda P., Pereverzeva M., Murray S.O. (2014). Pupil size reflects the focus of feature-based attention. J. Neurophysiol..

[12] Cheng M., Outerbridge J.S. (1975). Optokinetic nystagmus during selective retinal stimulation. Exp. Brain Res..

[13] Dubois M.F.W., Collewijn H. (1979). Optokinetic reactions in man elicited by localized retinal motion stimuli. Vis. Res..

[14] Howard I.P., Gonzalez E.G. (1987). Human optokinetic nystagmus in response to moving binocularly disparate stimuli. Vis. Res..

[15] Kanari K., Sakamoto K., Kaneko H. (2017). Effect of visual attention on the properties of optokinetic nystagmus. PLoS ONE.

[16] Kanari K., Kaneko H. (2019). Effect of visual attention and horizontal vergence in three-dimensional space on occurrence of optokinetic nystagmus. J. Eye Mov. Res..

[17] Kanari K. (2020). Pupil response is modulated by attention shift in optokinetic nystagmus. J. Opt. Soc. Am. A.

[18] Kanari K., Kaneko H. (2021). Pupil response is modulated with optokinetic nystagmus in transparent motion. J. Opt. Soc. Am. A.

[19] Naber M., Frässle S., Einhäuser W. (2011). Perceptual rivalry: Reflexes reveal the gradual nature of visual awareness. PLoS ONE.

[20] Konen C.S., Kleiser R., Seitz R.J., Bremmer F. (2005). An fMRI study of optokinetic nystagmus and smooth-pursuit eye movements in humans. Exp. Brain Res..

[21] Wu F., Zhao Y., Zhang H. (2022). Ocular autonomic nervous system: An update from anatomy to physiological functions. Vision.

[22] Kato I., Harada K., Hasegawa T., Igarashi T., Koike Y., Kawasaki T. (1986). Role of the nucleus of the optic tract in monkeys in relation to optokinetic nystagmus. Brain Res..

[23] Schiff D., Cohen B., Büttner-Ennever J., Matsuo V. (1990). Effects of lesions of the nucleus of the optic tract on optokinetic nystagmus and after-nystagmus in the monkey. Exp. Brain Res..

[24] Souto D., Kerzel D. (2021). Visual selective attention and the control of tracking eye movements: A critical review. J. Neurophysiol..

[25] Gamlin P.D., Zhang H., Clarke R.J. (1995). Luminance neurons in the pretectal olivary nucleus mediate the pupillary light reflex in the rhesus monkey. Exp. Brain Res..

[26] Hoffman J.E., Subramaniam B. (1995). The role of visual attention in saccadic eye movements. Percept. Psychophys..

[27] Kowler E., Anderson E., Dosher B., Blaser E. (1995). The role of attention in the programming of saccades. Vis. Res..

[28] Deubel H., Schneider W.X. (1996). Saccade target selection and object recognition: Evidence for a common attentional mechanism. Vis. Res..

[29] Mathôt S., van Der Linden L., Grainger J., Vitu F. (2015). The pupillary light response reflects eye-movement preparation. J. Exp. Psychol. Hum. Percept. Perform..

[30] Scala N.P., Spiegel E.A. (1940). Subcortical (passive) optokinetic nystagmus in lesions of the midbrain and of the vestibular nuclei. Confin. Neurol..

[31] Smith K.U., Bridgman M. (1943). The neural mechanisms of movement vision and optic nystagmus. J. Exp. Psychol..

[32] Arimoto K. (1958). Superior colliculus, as center of nystagmus. Wakayama Med. Rep..

[33] Brainard D.H. (1997). The psychophysics toolbox. Spat. Vis..

[34] Pelli D.G. (1997). The VideoToolbox software for visual psychophysics: Transforming numbers into movies. Spat. Vis..

[35] Matsuda K., Nagami T., Sugase Y., Takemura A., Kawano K. (2017). A widely applicable real-time mono/binocular eye tracking system using a high frame-rate digital camera. International Conference on Human-Computer Interaction.

[36] Einhäuser W., Stout J., Koch C., Carter O. (2008). Pupil dilation reflects perceptual selection and predicts subsequent stability in perceptual rivalry. Proc. Natl. Acad. Sci. USA.

[37] Frässle S., Sommer J., Jansen A., Naber M., Einhäuser W. (2014). Binocular rivalry: Frontal activity relates to introspection and action but not to perception. J. Neurosci..

[38] Tychsen L., Lisberger S.G. (1986). Visual motion processing for the initiation of smooth-pursuit eye movements in humans. J. Neurophysiol..

[39] Ferrera V.P. (2000). Task-dependent modulation of the sensorimotor transformation for smooth pursuit eye movements. J. Neurophysiol..

[40] Bergamin O., Kardon R.H. (2003). Latency of the pupil light reflex: Sample rate, stimulus intensity, and variation in normal subjects. Investig. Ophthalmol. Vis. Sci..

[41] Benjamini Y., Hochberg Y. (1995). Controlling the false discovery rate: A practical and powerful approach to multiple testing. J. R. Stat. Soc. Ser. B Methodol..

[42] Frattini D., Wibble T. (2021). Alertness and visual attention impact different aspects of the optokinetic reflex. Investig. Ophthalmol. Vis. Sci..

[43] van Die G.C., Collewijn H. (1986). Control of human optokinetic nystagmus by the central and peripheral retina: Effects of partial visual field masking, scotopic vision and central retinal scotomata. Brain Res..

[44] Hess E.H., Polt J.M. (1964). Pupil size in relation to mental activity during simple problem-solving. Science.

[45] Kahneman D., Beatty J. (1966). Pupil diameter and load on memory. Science.

[46] Steinhauer S.R., Siegle G.J., Condray R., Pless M. (2004). Sympathetic and parasympathetic innervation of pupillary dilation during sustained processing. Int. J. Psychophysiol..

[47] van der Wel P., van Steenbergen H. (2018). Pupil dilation as an index of effort in cognitive control tasks: A review. Psychon. Bull. Rev..

[48] Thompson H.S., Montague P., Cox T.A., Corbett J.J. (1982). The relationship between visual acuity, pupillary defect and visual field loss. Am. J. Ophthalmol..

[49] Johnson L.N., Hill R.A., Bartholomew M.J. (1988). Correlation of afferent pupillary defects with visual field loss on automated perimetry. Ophthalmology.

[50] Lowenstein O., Kawabata H., Loewenfeld I.E. (1964). The pupil as indicator of retinal activity. Am. J. Ophthalmol..

[51] Wang C.A., Boehnke S.E., White B.J., Munoz D.P. (2012). Microstimulation of the monkey superior colliculus induces pupil dilation without evoking saccades. J. Neurosci..

[52] Wang C.A., Munoz D.P. (2021). Coordination of pupil and saccade responses by the superior colliculus. J. Cogn. Neurosci..

[53] Krauzlis R.J. (2004). Recasting the smooth pursuit eye movement system. J. Neurophysiol..

